# 

**Published:** 1906-08-25

**Authors:** 


					the hospital
August 25th, 15)06.
TAIf
saorr Western Infirmary
rw^saorr Hester* Infirmary
No. 1,040. Vol XTj Roistered as"] SATURDAY,
- Nevrspaper.J
August 25th, 1906.
[Sabaaereippti3?7n4Terma] PRICE THREEPENCE

				

